# Transcriptional Sequencing Uncovers Survival Mechanisms of Salmonella enterica Serovar Enteritidis in Antibacterial Egg White

**DOI:** 10.1128/mSphere.00700-18

**Published:** 2019-02-13

**Authors:** Xiaozhen Huang, Xiujuan Zhou, Ben Jia, Nuo Li, Jingya Jia, Mu He, Yichen He, Xiaojie Qin, Yan Cui, Chunlei Shi, Yanhong Liu, Xianming Shi

**Affiliations:** a MOST-USDA Joint Research Center for Food Safety, School of Agriculture & Biology, and State Key Lab of Microbial Metabolism, Shanghai Jiao Tong University, Shanghai, China; b Department of Bioinformatics and Biostatistics, School of Life Sciences and Biotechnology, Shanghai Jiao Tong University, Shanghai, China; c Molecular Characterization of Foodborne Pathogens Research Unit, Eastern Regional Research Center, Agricultural Research Service, U.S. Department of Agriculture, Wyndmoor, Pennsylvania, USA; University of Kentucky

**Keywords:** DNA damage repair, alkaline pH adaptation, envelope damage repair, osmotic stress

## Abstract

The survival mechanism of Salmonella enterica serovar Enteritidis in antibacterial egg white is not fully understood. In our lab, an egg white-resistant strain, *S.* Enteritidis SJTUF 10978, was identified. Cell envelope damage and osmotic stress response (separation of cell wall and inner membrane as well as cytoplasmic shrinkage) of this strain surviving in egg white were identified through microscopic observation. RNA-Seq analysis of the transcriptome of *Salmonella* survival in egg white showed that a considerable number of genes involved in DNA damage repair, alkaline pH adaptation, osmotic stress adaptation, envelope damage repair, *Salmonella* pathogenicity island 2 (SPI-2), iron absorption, and biotin synthesis were significantly upregulated (fold change ≥ 2) in egg white, indicating that these pathways or genes might be critical for bacterial survival. RNA-Seq results were confirmed by qRT-PCR, and the survival analysis of six gene deletion mutants confirmed their importance in the survival of bacteria in egg white. The importance of alkaline pH adaptation and envelope damage repair for *Salmonella* to survive in egg white were further confirmed by analysis of *nhaA*, *cpxR*, *waaH*, and *eco* deletion mutants. According to the RNA-Seq results, we propose that alkaline pH adaptation might be the cause of bacterial osmotic stress phenotype and that the synergistic effect between alkaline pH and other inhibitory factors can enhance the bacteriostatic effect of egg white. Moreover, *cpxR* and *sigE* were recognized as the central regulators that coordinate bacterial metabolism to adapt to envelope damage and alkaline pH.

**IMPORTANCE** Salmonella enterica serovar Enteritidis is a major foodborne pathogen that causes salmonellosis mainly through contaminated chicken eggs or egg products and has been a worldwide public health threat since 1980. Frequent outbreaks of this serotype through eggs correlate significantly with its exceptional survival ability in the antibacterial egg white. Research on the survival mechanism of *S.* Enteritidis in egg white will help to further understand the complex and highly effective antibacterial mechanisms of egg white and lay the foundation for the development of safe and effective vaccines to prevent egg contamination by this *Salmonella* serotype. Key pathways and genes that were previously overlooked under bactericidal conditions were characterized as being induced in egg white, and synergistic effects between different antimicrobial factors appear to exist according to the gene expression changes. Our work provides new insights into the survival mechanism of *S.* Enteritidis in egg white.

## Abstract (precis)

Salmonella enterica serovar Enteritidis is a major foodborne pathogen that causes salmonellosis mainly through contaminated chicken eggs or egg products and has been a worldwide public health threat since 1980. Frequent outbreaks of this serotype through eggs correlate significantly with its exceptional survival ability in the antibacterial egg white. Research on the survival mechanism of *S.* Enteritidis in egg white will help to further understand the complex and highly effective antibacterial mechanisms of egg white and lay the foundation for the development of safe and effective vaccines to prevent egg contamination by this *Salmonella* serotype. Key pathways and genes that were previously overlooked under bactericidal conditions were characterized as being induced in egg white, and synergistic effects between different antimicrobial factors appear to exist according to the gene expression changes. Our work provides new insights into the survival mechanism of *S.* Enteritidis in egg white.

## INTRODUCTION

Salmonella enterica serovar Enteritidis is the predominant *Salmonella* serotype responsible for (90%) human salmonellosis resulting from the consumption of eggs or egg products and has become a global threat to public health (1 – 3). A serious outbreak of this serotype from table eggs occurred in 2010, leading to 1,939 reported illnesses and the recall of >500 million eggs in the United States (4). The survival ability of *S.* Enteritidis strains in the antibacterial egg white is significantly higher than that of other *Salmonella* serotypes, including *S.* Typhimurium as well as non-*Salmonella* strains such as Escherichia coli (5 – 7). This is an important factor leading to the spread and prevalence of internally infected eggs, which in turn has caused large-scale outbreaks of salmonellosis. These *S.* Enteritidis strains have been employed as model bacteria for studies on the antimicrobial activity of egg white (8).

Egg white is a complex antimicrobial environment due to its special physical and biochemical properties (9, 10). Its alkaline pH (9.3) disrupts pH homeostasis of neutrophiles and has been proven to be an antimicrobial factor (7, 11, 12). In addition, chelators in egg white, such as avidin and ovotransferrin, decrease the bioavailability of vitamins and iron and thus inhibit bacterial growth (9, 13, 14). Egg white also contains abundant antibacterial proteins and peptides that cause bacterial envelope damage. These include lysozyme, avian endogenous antimicrobial peptides such as β-defensins AvBD11, gallin (OvoDA1, OvoDB1), as well as small cationic peptides (CAMPs) derived from proteolytic hydrolysis of lysozyme and ovotransferrin (15 – 18). In addition, egg white contains potent DNase activity that kills bacteria through DNA damage (14).

The antimicrobial activity of egg white is affected by storage temperature and time. Elevated storage temperatures such as 37°C lead to a rapid increase in the rate of antimicrobial activity but result in a shorter half-life than with eggs stored at either 4°C or 20°C (19). Fresh table eggs stored at 37°C for 5 days exhibit the strongest anti-*Salmonella* activity (19). Inoculum level and incubation temperature also affect the antimicrobial activity of egg white. Higher incubation temperatures (>42°C) promote bacterial destruction (7, 12), and the ability of the bacteria to survive increases with inoculum size (12, 20). A compatible antibacterial model of egg white that includes high antibacterial activity and an appropriate inoculum size that can distinguish the survival advantage of this *Salmonella* serotype is needed to explore the specific survival mechanism of *S.* Enteritidis in this matrix.

So far, studies on the survival mechanisms of *S.* Enteritidis in the antibacterial egg white mainly focused on nutrient acquisition and defense against envelope damage in egg white (9). Certain genes involved in iron absorption (12), biotin synthesis (21), amino acid transport and metabolism (5), lipopolysaccharide metabolism (5, 21 – 23), cell wall integrity, cell motility- and stress-related regulators (5, 22, 24, 25), DNA repair (5, 14), and other important factors for *S.* Enteritidis survival in eggs have been identified by traditional genetic methods. Recently, transcriptome analysis of *S.* Enteritidis exposed to 10% egg white under bactericidal conditions (45°C) also identified induced genes involved in iron assimilation-, biotin synthesis-, and cell envelope damage-related pathways (8). However, the use of low egg white concentration (10%) may reduce its antibacterial ability, and high incubation temperature itself would place additional stress on bacteria. Thus, the bactericidal effect that resulted from the interaction between a high temperature and low egg white concentration may not fully reflect the transcriptional response of *Salmonella* surviving in egg white during egg storage.

In the current study, we examined the survival ability of *S.* Enteritidis strains in egg white using 80% egg white from specific-pathogen-free (SPF) eggs stored at 37°C for 4 to 5 days as a model system. We demonstrated the effect of initial inoculum size on bacterial survival, revealed the bactericidal mechanism of egg white by microscopic observation, and presented the global transcription profile of *S.* Enteritidis surviving in egg white through strand-specific RNA-Seq analysis. Critical metabolic pathways and genes that promote bacterial survival in this matrix were also identified.

## RESULTS

### Effect of inoculum size on survival ability of *Salmonella* strains of different serotypes in egg white.

**FIG 1:**
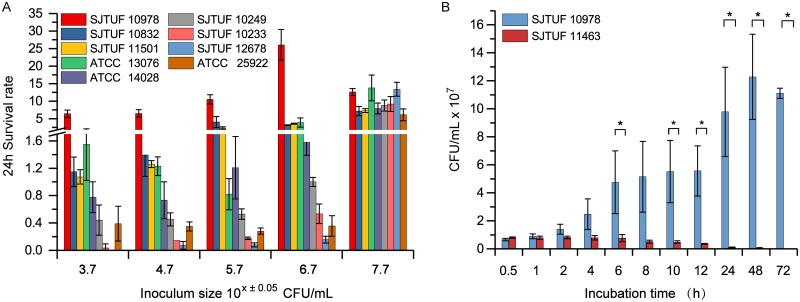
Growth of *Salmonella* and E. coli in egg white. (A) Comparison of initial inoculum size on 24-h survival rates of the listed strains in egg white at 37°C. *S.* Enteritidis strains (SJTUF 10978, SJTUF 10832, SJTUF 11501, and ATCC 13076), *S.* Typhimurium (ATCC 14028, SJTUF 10249, and SJTUF 10233), *S.* Indiana SJTUF 12678, and E. coli ATCC 25922 were tested. (B) Survival at 37°C in egg white of strains SJTUF 10978 and SJTUF 11463 using an initial inoculum of 5 × 10^6^ ± 2 × 10^6^ CFU/ml. Values that are significantly different (*P < *0.05) are indicated by a bar and asterisk.

In order to optimize the antibacterial model of egg white at 37°C, we tested the effect of different inoculum concentrations on bacterial survival ability in egg white. We tested five inoculum concentrations from 10^3^ to 10^7^ CFU/ml for four *S.* Enteritidis strains, three *S.* Typhimurium strains, and one inoculum concentration each for *S.* Indiana and E. coli strains. When the bacterial inoculum was <7 log CFU/ml, the survival rates of *S.* Enteritidis strains (except for strain ATCC 13076) at 24 h were higher than any other tested strains (Fig. 1A). When the inoculum level was >7 log CFU/ml, the egg white had no antibacterial activity toward any tested bacteria at 37°C, and there were no differences among various serotypes in survival ability. Thus, the survival advantage of *S.* Enteritidis existed only when the inoculum size was below 7 log CFU/ml. Therefore, an initial inoculum size of 6.7 log CFU/ml was used for the following experiments.

We compared the growth trends of the resistant strain SJTUF 10978 and the sensitive strain SJTUF 11463 in egg white. Strain SJTUF 10978 could overcome the limitation of egg white and reached 10^8^ CFU/ml at 24 h, while strain SJTUF 11463 was gradually killed. At 6 h, 12 h, and 24 h, the bacterial viability of the two strains became significantly different (*P* < 0.05) (Fig. 1B).

### Microscopic observation of bacterial damage in egg white.

**FIG 2:**
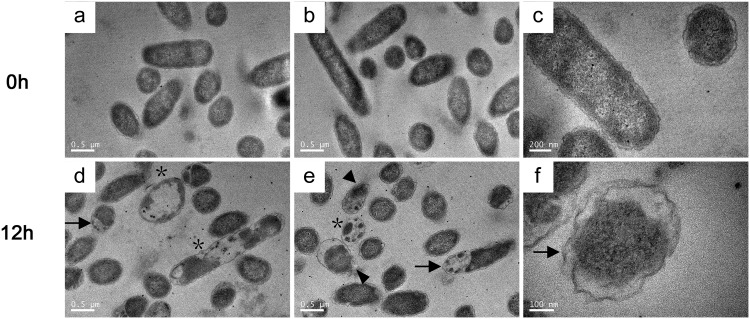
TEM observation of the bacterial damage caused by egg white. (a to c) Logarithmic-growth-phase culture of SJTUF 10978 in M9FeS as the 0-h sample; (d and e) SJTUF 10978 cultures exposed to egg white for 12 h as indicated. Cytoplasmic vacuoles (asterisks) and examples of cytoplasmic shrinkage or separation between the cell wall and membrane (arrows) and of cell wall and membrane disruption (arrowheads) are shown.

In this study, bacterial cell injury induced by egg white was observed with transmission electron microscopy (TEM). In the TEM photographs, the bacterial wall and membrane of strain SJTUF 10978 at 0 h (logarithmic phase grown culture in M9FeS medium) was intact, and the shallow chromatin area was clearly visible (Fig. 2a to c). After exposure to egg white for 12 h, there was separation of the cell wall and inner membrane as well as cytoplasmic shrinkage (Fig. 2d to f, black arrows). This indicates that the bacteria had undergone hypertonic stress in egg white. Some bacteria showed internal vacuoles, blurred cell wall edges, and disrupted cell walls, indicating the presence of damage to cell wall and membrane and the leakage of intracellular contents (Fig. 2d to f, black asterisks and arrowheads).

### Overview of RNA-Seq results.

The egg white-resistant strain *S.* Enteritidis SJTUF 10978 was used for strand-specific RNA sequencing after exposure to egg white for 6, 12, and 24 h. These samples were compared to logarithmic-phase samples of strain SJTUF 10978 cultured in M9FeS (0-h sample) to calculate the number of differentially expressed genes (DEGs). Genes with FPKM (fragments per kilobase of transcript per million mapped fragments) values of <30 were removed from subsequent analysis. Using this criterion, M9FeS and egg white cultivation resulted in 3,082 to 3,205 expressed genes accounting for 65% to 68% of the whole genome. In this group, 1,200 to 1,415 genes had significant levels of log_2_ (fold change) ≥ 1 (*P*_adj_ <0.05) compared to the control. These genes accounted for 25.4 to 29.9% of the whole genome, indicating a large shift in the transcriptional response to egg white (Table 1; see also Data Set S1 for a list of the DEGs).

**TABLE 1:** Analysis of expressed and differentially expressed genes in the chromosome plus plasmid (4,733 coding sequences)

Sample	No. of genes with an FPKM ≥ 30	Ratio (%)^*a*^	No. of DEGs^*b*^	No. of genes	
				Upregulated	Downregulated
0 h	3,145	66.44			
6 h	3,082	65.12	1,200	512	688
12 h	3,133	66.19	1,312	593	719
24 h	3,205	67.72	1,415	655	760

a The ratio of genes with an FPKM ≥ 30 to all genes in the whole genome.

b Compared with 0-h samples, genes with a log_2_ (fold change) ≥ 1 and *P*_adj_ < 0.05 were identified as differentially expressed genes (DEGs).

10.1128/mSphere.00700-18.1 DATA SET S1 DEGs of *S.* Enteritidis SJTUF 10978 exposed to egg white for the indicated times. Download Data Set S1, XLSX file, 0.7 MB. Copyright © 2019 Huang et al. 2019 Huang et al. This content is distributed under the terms of the Creative Commons Attribution 4.0 International license.

**FIG 3:**
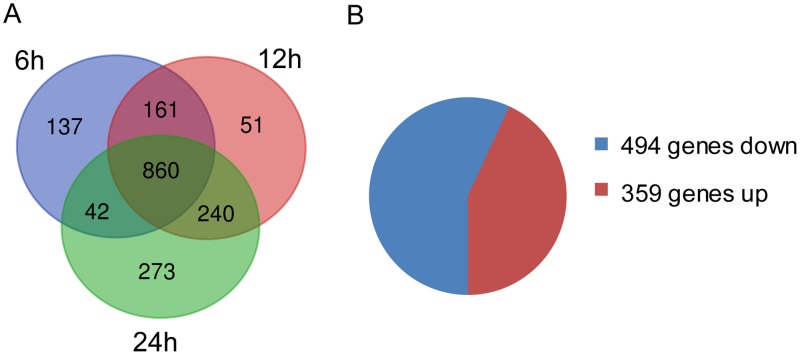
Overview of differentially expressed genes (DEGs) at 6-, 12-, and 24-h incubations in the presence of egg white. (A) DEGs of *S.* Enteritidis SJTUF 10978 exposed to egg white. (B) DEGs showing similar variations.

We found that 860 genes were significantly changed in all three egg white incubation samples (6, 12, and 24 h) compared with the control (Fig. 3A). Of these genes, 359 genes were commonly upregulated and 494 genes were commonly downregulated at all three time points such that 853 genes presented the same trend in variation over time after exposure to egg white (Fig. 3B). This accounts for 71.1, 65.0, and 60.3% of the total DEGs at the three time points (6, 12, and 24 h), respectively. Therefore, the bacterial transcriptomes of the egg white incubation samples were more similar to each other than with the M9FeS growth samples.

**FIG 4:**
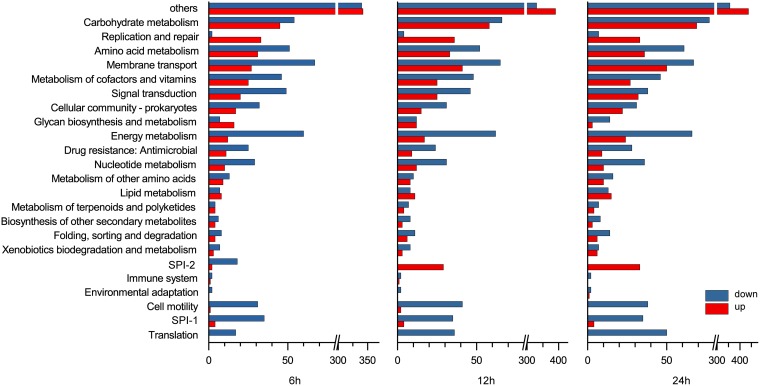
KEGG cluster of DEGs of *S.* Enteritidis SJTUF 10978 exposed to egg white for the indicated times. Functional categories for genes associated with replication and repair and SPI-1 and SPI-2 genes were partially collected according to reference 89. The numbers of DEGs are shown on the *x* axes. (The DEG members are listed in Data Set S1 in the supplemental material).

By KEGG and GO cluster analysis, we found significantly upregulated pathways and genes that were not identified by a previous study under bactericidal condition (8), including DNA replication and repair, alkaline pH adaptation, peptidoglycan synthesis, and *Salmonella* pathogenicity island 2 (SPI-2). Consistent with previous data under bactericidal conditions, we found upregulated genes involved in osmotic stress, cell envelope repair, iron acquisition, and biotin synthesis and significantly downregulated genes involved in energy metabolism, translation, cell motility, and SPI-1 pathways (Fig. 4) (8). The downregulation of these genes may be the result of changes in culture conditions and decrease in growth rate. Here, we focused on stress-related genes that were upregulated.

### Verification of RNA-Seq results by qRT-PCR.

**FIG 5:**
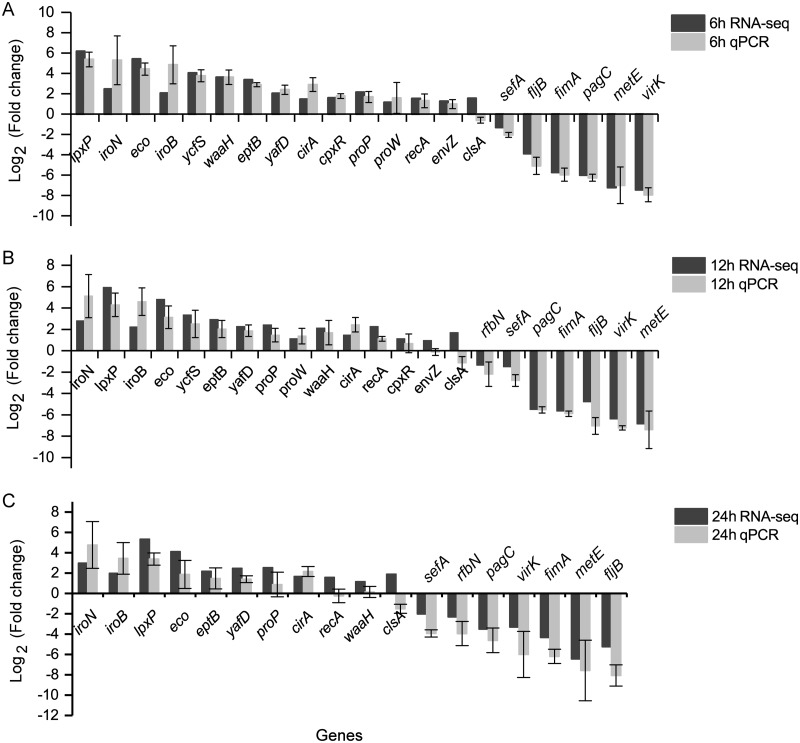
Verification of RNA-Seq results by RT-qPCR. (A to C) Comparisons between RT-qPCR and RNA-Seq results with RNA samples exposed to egg white for 6 h (A), 12 h (B), and 24 h (C). Mean values and standard errors of the means (error bars) from three independent experiments are indicated.

To verify the RNA-Seq results, qRT-PCR assays were performed on 18 to 21 genes with 16S rDNA as the reference gene. The qRT-PCR results were highly consistent with the RNA-Seq results for all three time points (Fig. 5A to C). The consistency levels were 95, 90.5, and 77.8% at the three time points. This result confirmed the reliability of the RNA-Seq results.

### Survival capacity of deletion mutants in egg white.

**FIG 6:**
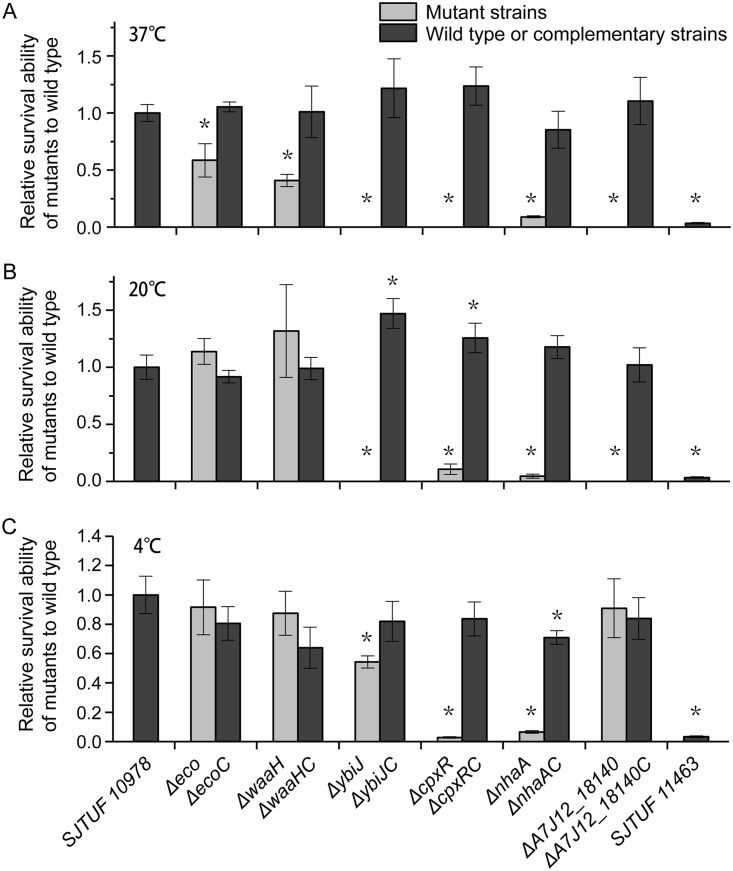
Comparison of egg white survival capacity of strain SJTUF 10978 and its derivatives. SJTUF 10978 and its six DEG mutant and complementary strains were incubated in egg white stored at 37°C for 1 day (A) and with egg white stored at 20°C (B) and 4°C (C) for 5 days. The relative survivability of derivatives relative to the wild type is shown on the *y* axes. Mean values are taken from three independent experiments, and the standard errors of the means (error bars) are shown. *, *P ≤ *0.05.

In order to determine whether the identified DEGs had any effects on the viability of bacteria in egg white, six genes that were significantly upregulated in egg white were deleted individually in the SJTUF 10978 genome using the λ Red recombination system, and complementary strains were constructed using sucrose suicide plasmid pRE112 (see Table S1 in the supplemental material) (26). The six genes were *eco* (encoding ecotin), *waaH* (encoding glycosyltransferase), *nhaA* (encoding Na^+^/H^+^ antiporter), *cpxR* (encoding response regulator), *ybiJ* (encoding uncharacterized protein), and *A7J12_18140* (encoding uncharacterized protein). No general growth defect of these deletion mutants was found when cultured either in LB or M9 medium except for the *ΔnhaA* mutant in M9 medium (Fig. S1). The *ΔnhaA* mutant exhibited a lower growth density during the stationary phase in M9 medium (Fig. S1). The survival ability of these gene deletion mutants and complementary strains was tested in egg white stored at 37°C (lab storage), 20°C (industrial egg storage temperature), and 4°C (refrigerator temperature). All six gene deletion mutants had significant viability decreases after exposure to egg white stored at 37°C for 24 h compared to the wild-type strain SJTUF 10978 (Fig. 6A). When incubated with egg white stored at 20°C, the survival rates of four gene deletion mutants (*ΔnhaA*, *ΔybiJ*, *ΔcpxR*, and *ΔA7J12_18140*) started to reduce after 2 days of incubation (data not shown) and reached the same level as their survival rate at 37°C（for 24 h）after incubation for 5 days (Fig. 6B). When incubated with egg white stored at 4°C, only three gene deletion mutants (*ΔnhaA*, *ΔcpxR*, and *ΔybiJ*) showed significant reduction after 5 days of incubation (Fig. 6C). The survival abilities of the complementary strains of these six gene mutants were restored to the level of the wild type at three storage temperature conditions, with the exception that the *ΔnhaAC* mutant was 29% lower than the wild type when incubated with egg white stored at 4°C (Fig. 6C). These results indicated that these genes may play key roles for the survival of *Salmonella* strain SJTUF 10978 in egg white, and most of the DEGs that were explored with egg white stored at 37°C also applied to egg white stored at 20°C, which may be due to the antimicrobial ability of egg white stored at 37°C for 5 days being equal to that of egg white stored at 20°C for 12 days (19). Our results suggested that the DEGs from the RNA-Seq results were closely related to the resistance phenotype of SJTUF 10978 in egg white. These genes can be used to dissect critical pathways important for bacterial adaptation in the antibacterial egg white.

10.1128/mSphere.00700-18.8 FIG S1 Growth curves of gene deletion mutants and complementary strains in LB and M9 medium. (A) Growth curves of gene deletion mutants and complementary strains in LB. (B) Growth curves of gene deletion mutants and complementary strains in M9 medium. Mean values are taken from three independent experiments, and the standard errors of the mean are shown. Download FIG S1, TIF file, 3.0 MB. Copyright © 2019 Huang et al. 2019 Huang et al. This content is distributed under the terms of the Creative Commons Attribution 4.0 International license.

10.1128/mSphere.00700-18.9 TABLE S1 Strains and plasmids used in this study. Download Table S1, DOCX file, 0.02 MB. Copyright © 2019 Huang et al. 2019 Huang et al. This content is distributed under the terms of the Creative Commons Attribution 4.0 International license.

### DNA damage repair and SOS response.

**FIG 7:**
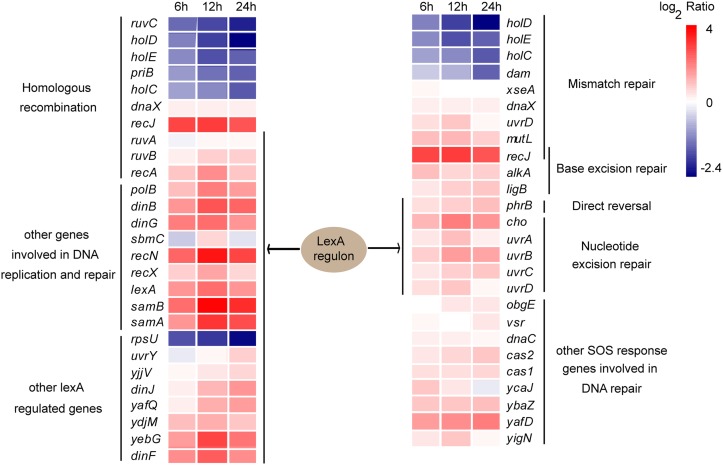
DNA damage repair and SOS response of *S.* Enteritidis exposed to egg white. LexA regulon genes were collected from RegulonDB (http://regulondb.ccg.unam.mx/) and RegPrecise (http://regprecise.lbl.gov/).

Forty genes that significantly changed in at least one sampling point were found to be involved in DNA replication and repair in either SOS-dependent or SOS-independent modes. Of these genes, 32 genes were induced, which accounted for 80% of the DEGs associated with DNA replication and repair (Fig. 7; see also Data Set S2 for a list of the genes and references). Regulators of the SOS response, including *lexA*, *recA*, *recX* as well as 26 genes of the LexA regulon were all increased after exposure to egg white (Fig. 7). This indicates the activation of the SOS response and 18 genes of the induced genes have been proven to have functions in DNA replication and repair (27).

10.1128/mSphere.00700-18.2 DATA SET S2 List of DNA replication-, DNA repair-, and SOS response-related DEGs. Download Data Set S2, XLSX file, 0.02 MB. Copyright © 2019 Huang et al. 2019 Huang et al. This content is distributed under the terms of the Creative Commons Attribution 4.0 International license.

Multiple DNA repair pathways were also activated by exposure to egg white. These DNA repair pathways include the following: (i) DNA repair by reversal of DNA damage photolyase-encoding gene *phr*; (ii) DNA single-strand damage repair genes *uvrABCD* and *cho*, mismatch repair genes *dnaX*, *mutL*, *uvrD*, *recJ*, and *vsr*, base excision repair genes *alkA*, *ligB*, and *recJ*; (iii) homologous recombination genes required for DNA double-strand damage repair *ruvB*, *recA*, *recJ*, *recNI*, and *dinG*; and (v) DNA translesion synthesis DNA polymerase II, IV, and V homologue SamAB genes *polB*, *dinB*, and *samAB*. All were significantly upregulated 2- to 13.5-fold at one or more sampling points (Fig. 7; Data Set S2).

In addition to the above pathways, induced genes that are involved in rescuing stalled or collapsed replication forks after DNA damage were also identified. These genes included the GTPase *obgE* gene and the DNA-dependent ATPase *ycaJ* gene. Meanwhile, the DNA replication proteins encoded by *dnaC* and *sbmC* that protect cells against DNA damage resulted from alkylating agents and the supercoiling activity of DNA gyrase, a DNA base-flipping protein *ybaZ* gene that recognizes and flips damaged bases out of DNA duplexes for repair were also induced. The endonuclease/exonuclease/phosphatase family member *yafD* and *cas1*, *cas2*, and *yigN* encoding a putative recombinase with LexA-dependent expression were all upregulated in egg white.

Six LexA regulon genes that do not have defined functions in DNA repair were also significantly induced in egg white (Fig. 7). They are involved in carbon metabolism (*uvrY*), toxin-antitoxin module (*yafQ/dinJ*, *dinF*) or uncharacterized (*yebG*), respectively.

### Stress response to alkaline pH.

The RNA-Seq results indicated that pH homeostasis of strain SJTUF 10978 in egg white was achieved by increasing the expression of genes involved in cation/proton antiporters, amino acid dehydrogenases/deaminases, and carbonic anhydrase, meanwhile decreasing the expression of the proton pumping respiratory chain complex (Data Set S3 for a list of the genes and references). The upregulated cation/proton antiporters decreased intracellular pH by pumping in protons (28). For example, the Na^+^/H^+^ antiporter-encoding *nhaA* gene (2H^+^:1Na^+^), the primary antiporter for alkaline pH adaptation and its transcriptional activator *nhaR* and *rpoS* (29, 30) were upregulated by 3.6- to 10-fold during incubation. When *nhaA* was deleted in the genome, relative survivability of *Salmonella* in egg white dropped to less than 9% compared to the wild type (Fig. 6). Thus, we genetically confirmed that *nhaA* and alkaline pH adaptation were essential for bacterial adaptation to the egg white environment. In addition, the expression of cation/proton antiporter gene *chaA*, the K^+^/H^+^ antiporters *nhaP2* (*cvrA*), and the glutathione-gated K^+^ efflux system *kefGB* which imported protons to the cytoplasm coupled with cation efflux, were also significantly increased in egg white. The DedA/Tvp38 family member *yqjA* plays a role in E. coli growth under alkaline conditions. This gene was upregulated across the incubation time course. Amino acid deaminases can also reduce intracellular pH by the outward diffusion of NH_3_ while retaining an acidic cytoplasmic product (31). The *sdaA*, *gdhA*, *dadA*, and *tdh* genes that encode L-serine dehydratase, glutamate dehydrogenase, D-amino acid dehydrogenase, and L-threonine 3-dehydrogenase, respectively, would catalyze production of ammonia and generate metabolic acids. These genes were all upregulated in egg white. The *sdaA* and *gdhA* genes are also essential for E. coli to adapt to alkaline pH (32). The β-carbonic anhydrase encoded by *yadF* can buffer intracellular pH by catalyzing CO_2_ and water interconversion to bicarbonate and protons at alkaline pH (33). This gene was highly expressed (FPKM > 3,900) and induced by twofold. The expression of proton pumping respiratory chain complex NADH-ubiquinone oxidoreductase (*nuo*) was decreased to minimize proton loss from the cytoplasm during proton motive force generation.

10.1128/mSphere.00700-18.3 DATA SET S3 List of alkaline pH stress adaptation-related DEGs. Download Data Set S3, XLSX file, 0.01 MB. Copyright © 2019 Huang et al. 2019 Huang et al. This content is distributed under the terms of the Creative Commons Attribution 4.0 International license.

### Osmotic stress response.

Genes involved in osmoprotectant synthesis, transport, and some osmotically inducible proteins were upregulated in egg white (Data Set S4). The osmoprotectant transporter ProP (*proP*) and parts of another ABC-type transporter ProU (*proW* and *proV*) (34) were significantly upregulated in egg white. Because of cotransport protons and osmoprotectants, the upregulation of *proP* might also contribute to alkaline pH adaptation. On the other hand, trehalose synthesis and utilization genes (*otsA* and *otsB* and *treA* and *treF*) were also significantly induced in egg white (34).

10.1128/mSphere.00700-18.4 DATA SET S4 List of osmotic stress response-related DEGs. Download Data Set S4, XLSX file, 0.01 MB. Copyright © 2019 Huang et al. 2019 Huang et al. This content is distributed under the terms of the Creative Commons Attribution 4.0 International license.

In addition, the expression of a lipoprotein gene *nlpI* and osmotically inducible protein *osmB* were induced. The *osmB* and *nlpI* genes help maintain membrane stability during osmotic pressure fluxes and facilitate membrane resealing (35). Thus, the upregulation of these two genes may promote the integrity of the cell membrane in the egg environment.

### Bacterial envelope stress responses to egg white.

**FIG 8:**
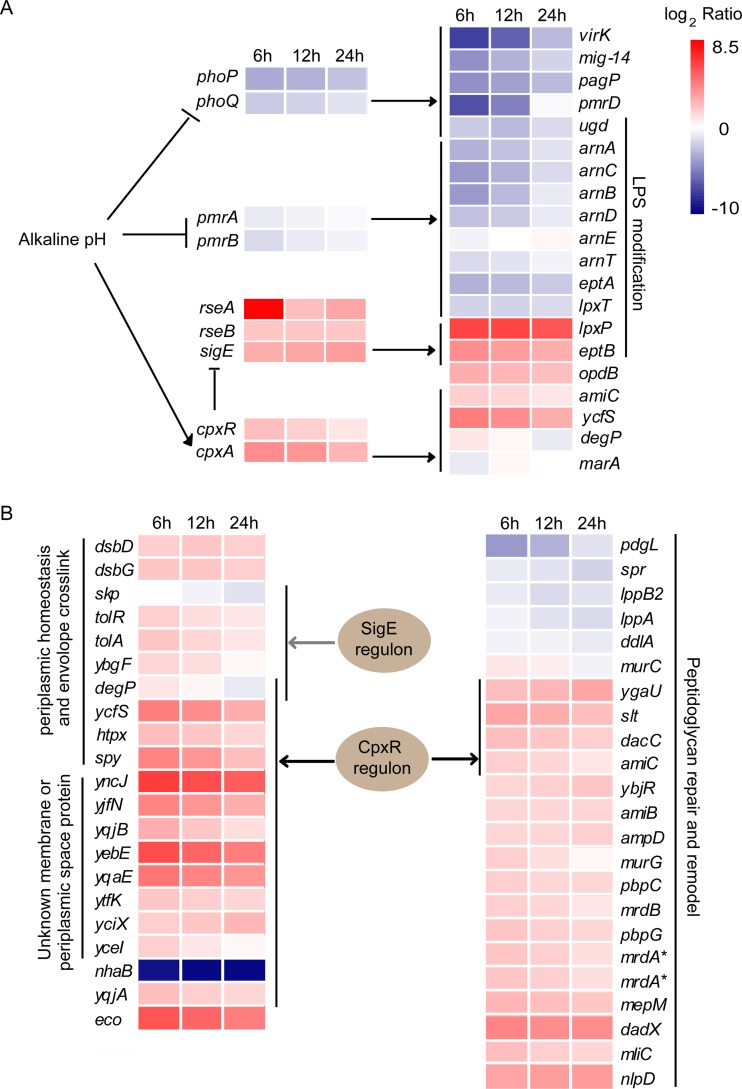
Envelope stress responses of Salmonella Enteritidis exposed to egg white. (A) DEGs and pivotal regulatory factors enriched in the CAMP resistance pathway. (B) DEGs and pivotal regulatory factors involved in periplasmic homeostasis, peptidoglycan repair, and remodel and envelope cross-linking. The asterisks indicate that *A7J12_02965* and *A7J12_05665* are functionally similar to *mrdA*.

RNA-Seq results also revealed that bacteria combat cell envelope stress induced by egg white by increasing the expression of genes involved in periplasmic homeostasis, envelope cross-linking, and peptidoglycan synthesis and degradation pathways (Fig. 8B; see Data Set S5 for a list of the genes and references). Genes involved in folding and degradation of periplasmic proteins and envelope cross-linking were induced in egg white (Fig. 8B; see Data Set S5 for a list of the genes and references), including periplasmic chaperone *spy*, two components of the periplasmic reducing system *dsbG* and *dsbD*, as well as membrane-localized protease gene *htpX* which plays a role in proteolytic quality control of membrane proteins in conjunction with FtsH (36). The *ycfS* gene that catalyzes anchoring of the Braun lipoprotein to peptidoglycan (PG) was specifically induced by 6- to 16-fold in egg white. In egg white, *ybgF*, *tolR*, and *tolA* were significantly induced at 6 h, and these genes were part of the *ybgC*-*ybgF*-*tolQRAB*-*pal* cluster. This cluster of genes encodes the Tol-Pal complex (peptidoglycan-associated lipoprotein) and is involved in maintaining cell envelope stability. The increase of Braun lipoprotein, PG cross-linking, and Tol-Pal complex made different layers of the cell envelope more stable in egg white (37 – 39).

10.1128/mSphere.00700-18.5 DATA SET S5 List of envelope stress response-related DEGs. Download Data Set S5, XLSX file, 0.02 MB. Copyright © 2019 Huang et al. 2019 Huang et al. This content is distributed under the terms of the Creative Commons Attribution 4.0 International license.

In addition, the protease inhibitor ecotin-encoding *eco* gene was highly induced by 17- to 43-fold in egg white. Deletion of *eco* reduced the survival rate of bacteria by 40% at 37°C (Fig. 6A). It can protect bacteria from an exogenous target neutrophil elastase (40), which can kill bacteria by degradation of outer membrane protein A.

Peptidoglycan repair and remodeling were also enhanced in the presence of egg white. Peptidoglycan precursor biosynthesis-related genes, including the racemase *dadX* and glycosyltransferase *murG* genes, were significantly upregulated in egg white. In addition, genes in the peptidoglycan biosynthesis pathway increased. For example, the expression of three endopeptidase genes (*pbpG*, *yebA*, and *dacC*), one lytic transglycosylase gene (*slt*), four amidase genes (*amiC*, *amiB*, *amiD*, and *ampD*), murein hydrolase activator *nlpD*, and peptidoglycan polymerase *mrdB*, which is responsible for cell wall elongation, all increased during incubation with egg white. On the other hand, genes involved in PG cross-linking were induced in egg white. The D,D-transpeptidases encoded by *mrdA* (*A7J12_02965* and *A7J12_05665*) that catalyze primary cross-linking of PG with D-Ala^4^-meso-DAP^3^ were upregulated. The L,D-transpeptidase *ycbB* involved in synthesis of *meso*-DAP^3^-*meso*-DAP^3^ peptidoglycan cross-links (41) increased slightly (1.6- to 1.7-fold) in egg white. The *ygaU* gene that assists in increasing 3-3 cross-links (*meso*-DAP^3^-*meso*-DAP^3^) in an unknown way was induced across the incubation time course. Interestingly, *mliC*, encoding a lysozyme inhibitor of MliC family, was induced significantly and might contribute to resistance to lysozyme.

### Cationic antimicrobial peptide resistance pathway.

DEGs involved in the cationic antimicrobial peptide (CAMP) resistance pathway were highly enriched (*P* < 0.05) at 6 and 12 h in egg white (Fig. 8A and Data Set S6). The RNA-Seq results indicated that expression of the two-component system (TCS) involved in CAMP resistance was downregulated significantly. The TCS systems *phoP*/*phoQ* and *pmrA*/*pmrB* as well as their target genes *mig-14*, *virK*, *pagP*, *arnABCDT*, *pmrD*, and *eptA*, which enhance resistance to cationic peptides by CAMP binding and efflux (42) or by modifying lipopolysaccharide (43, 44) decreased significantly. The only alkaline pH-induced TCS involved in the CAMP resistance pathway was *cpxR/cpxA*, and these two genes are critical for the TCS response to cell envelope stress (45). The TCS *cpxR*/*cpxA* and part of its regulon *amiC* and *ycfS* (46) act to maintain cell envelope integrity and contribute to CAMP resistance (47) (Fig. 8A). This indicates that *cpxR/cpxA* became the key regulator of CAMP resistance in egg white. Another section of the *cpxR*/*cpxA* regulon associated with CAMP resistance—multidrug efflux pump *acrAB*-*tolC*, the peripheral chaperones *dabA*, *degP*, and *ppiA* (48, 49) were either constitutively expressed or repressed in egg white. This may be due to decreased growth or other complex factors.

10.1128/mSphere.00700-18.6 DATA SET S6 DEGs of cationic antimicrobial peptide (CAMP) resistance pathway and upregulated DEGs regulated by SigE. Download Data Set S6, XLSX file, 0.02 MB. Copyright © 2019 Huang et al. 2019 Huang et al. This content is distributed under the terms of the Creative Commons Attribution 4.0 International license.

Lipopolysaccharide (LPS) modification is an important mechanism to resist CAMPs by decreasing the negative charge of the bacterial surface and increasing hydrophobicity of the outer membranes (42). Although part of the modification was inhibited due to the suppression of the main regulatory TCS *pmrA*/*pmrB* (50), four genes that participate in LPS modification were significantly changed (Fig. 8A). The *eptB* and *waaH* genes are responsible for phosphoethanolamine modification and glucuronic acid modification of LPS, respectively (51, 52). These genes were both induced in egg white. The two modifications would reduce the negative charge and increase the positive charge of lipopolysaccharide (51, 52) and reduce CAMP cell surface adsorption potential (53). Consistent with the RNA-Seq results, mutation of the *waaH* gene significantly reduced the survival ability of SJTUF 10978 in egg white by 60% at 37°C (Fig. 6A). In addition, the expression level of *lpxT*, a gene that transfers a phosphate group to lipid A to form lipid A 1-diphosphate, decreased significantly. The inhibition of LpxT-dependent phosphorylation of lipid A also promotes resistance to antimicrobial peptides by reducing the negative charge of LPS (54). The *lpxP* gene responsible for palmitoylation of lipid A at low temperatures (55) was induced in egg white. However, whether it would generate CAMP resistance to bacteria by palmitoylation of lipid A like gene *pagP* (43) is unknown.

The cytosolic oligopeptidase B-encoding *opdB* gene enhances bacterial resistance to several proline-rich antimicrobial peptides by proteolytic cleavage of antimicrobial peptides (56). This gene was specifically induced in egg white three- to fourfold and may play an important role in blocking antimicrobial peptides that invade the cytoplasm.

### *Salmonella* pathogenicity islands.

In our data, we found that 35/43 genes belonging to SPI-1 were significantly decreased across the incubation period. This result is consistent with previous data (8). Moreover, we found that 34/44 genes located on SPI-2 were significantly changed in egg white. Of these genes, 18 genes involved in type III secretion decreased at 6 h. All 34 genes were upregulated by 1.7- to 5.6-fold at 12 h and highly induced by 2- to 53-fold at 24 h (Fig. 4).

## DISCUSSION

In this study, we found that an inoculum size below 10^7^ CFU/ml was necessary to investigate the survival advantage of *S.* Enteritidis in egg white compared with other *Salmonella* serotypes and E. coli. We combined an inoculum level of 10^6^ CFU/ml with microscopic observation, RNA-Seq analysis, and gene deletion to reveal key pathways and genes that help bacteria to survive in this niche.

### Bacteria employ multiple DNA repair mechanisms to fight against DNA damage in egg white.

Egg white contains potent nuclease activity (14), which will cause DNA damage once the cell envelope is damaged. In egg white, bacteria employed both SOS-dependent and SOS-independent DNA repair processes to fight against DNA damage (Fig. 7; see also Data Set S2 in the supplemental material). These processes include DNA direct reverse repair, nucleotide excision repair, mismatch repair, base excision repair, homologous recombination, and DNA translesion synthesis. DNA translesion synthesis polymerases DNA polymerase IV and SamAB restart replication in an error-prone manner (57, 58). The continuous upregulation of these polymerases indicated that bacterial DNA damage persisted in egg white, and the lesion was so serious that it was beyond the capability of general high-fidelity repair; as a result, mutational repair mechanisms were induced to tolerate the damage (27). On the other hand, this suggests that egg white is an environment that tends to induce gene mutations in bacteria. The *lexA* and *recA* genes encode key regulators of the SOS response, and 19 upregulated genes that associated with DNA replication repair belong to the LexA regulon, suggesting that these two genes also play key roles in DNA damage repair caused by bacteria against egg white.

The *yafD* nuclease gene was upregulated by fourfold in egg white. This was consistent with the previous finding that *yafD* deletion significantly reduces bacterial viability in egg white, while overexpression of this gene significantly enhances bacterial viability in this matrix (14). However, no DNA replication and repair genes were significantly changed in previous microarray results that included 10% egg white at 45°C (8), which may be due to differences in the egg white model.

In summary, our data revealed the bacterial DNA damage repair mechanisms in egg white at the transcriptional level, indicating that DNA damage repair and tolerance are crucial for the viability of bacteria in egg white.

### Alkaline pH adaptation is critical for survival.

The alkaline pH of egg white would cause stress in *Salmonella* by affecting the normal functioning of the cytoplasm as well as the transmembrane proton motive force and energy production (28, 59). Artificially changing the pH of egg white decreases the bactericidal effect (7, 19). Using a *nhaA* gene deletion, we demonstrated that alkaline pH adaptation is crucial for *S.* Enteritidis to survive in egg white (Fig. 6). Bacteria adapt to alkaline pH by upregulation of genes encoding cation/antiporter, amino acid deaminases, and carbonic anhydrase to acidify the cytoplasm and downregulation of the expression of the proton pump *nuo* genes to reduce proton efflux (Data Set S3). However, we did not observe any change of the phage shock protein cluster (*pspABCDEF*) that was required for survival at high pH. These genes were induced in previous microarray experiments (8).

In addition to acting alone, alkaline pH can also work synergistically with other antibacterial factors. We propose that the alkaline pH environment can enhance the bacterial sensitivity to CAMP by inhibiting the expression of CAMP resistance-related genes and may reduce the intracellular osmotic pressure due to efflux of cations, thereby increasing bacterial susceptibility to osmotic stress (discussed below).

### Bacterial osmotic stress responses do exist in egg white.

For the first time, we observed obvious cytoplasm shrinkage and separation of cell wall and the inner membrane of strain SJTUF 10978 exposed to egg white for 12 h (Fig. 2) by TEM, thus confirming that bacteria do experience hyperosmotic stress in egg white. This conclusion is consistent with the upregulation of hyperosmotic stress-related genes in egg white in the study of Baron et al. (8) and our transcriptomic results (Data Set S4).

However, the osmolarity of egg white (240 mosm/liter) is comparable to that of TSB culture medium (320 mosm/liter) (9). Thus, egg white should not be a hyperosmotic environment. The reason for a hyperosmotic response in a nonhyperosmotic environment may be explained by alkaline pH adaptation. Cation/proton antiporters import protons and export cations to reduce intracellular pH. This may reduce the ion concentration in the cytoplasm, leading to a decrease in the cytoplasmic osmotic pressure. If the descending osmotic pressure cannot be offset by the osmoprotectant transport or synthetic system, a lower cytoplasm osmotic pressure would form compared with that of egg white. This is possible because transport of osmoprotectant requires energy or cotransport with protons (60, 61); however, in egg white, the expression of bacterial energy-producing systems greatly decreased (Fig. 4), and protons may be limited in the alkaline pH. This hypothesis is consistent with the fact that in an alkaline pH environment, E. coli grew better in media with higher salt concentrations or osmotic pressure (62, 63).

Thus, we confirmed that bacteria do experience hyperosmotic stress in egg white through microscopic observations and RNA-Seq data. We propose that the alkaline pH adaptive process may be the cause of the osmotic pressure imbalance between bacteria and the egg white environment.

### Multiple pathways respond to envelope damage.

Egg white possesses multiple antimicrobial factors targeting the envelope including alkaline pH, lysozyme, proteases, and CAMPs. Correspondingly, we found evidence of disruption of the cell wall/membrane for bacteria through TEM observation, indicating that envelope damage is an important antibacterial mechanism for egg white. This result is consistent with experiments performed with E. coli under bactericidal conditions (45°C, 10% egg white) (64). Our RNA-Seq data revealed that bacteria maintained envelope integrity by upregulating genes involved in maintaining periplasmic space homeostasis, enhancement of envelope cross-linking, peptidoglycan synthesis and remodeling, and modifying LPS surface charge.

The peptidoglycan layer is an important physical barrier for protection against both mechanical and osmotic stresses and is the target for egg white lysozyme. Although the replication rate of *S.* Enteritidis decreased significantly in egg white, the PG biosynthesis and cross-linking related genes were significantly upregulated (Fig. 7B). The *meso*-DAP^3^-*meso*-DAP^3^ cross-links are formed in response to a variety of stress conditions (65 – 67) and thought to help stabilize the bacterial envelope by reinforcing the cell wall (68, 69). These suggest that increased PG thickness or repair occurred during incubation with egg white to combat lysozyme attacks. However, upregulation of PG synthesis genes was not observed in egg white under bactericidal conditions (8), indicating different physiological responses of bacteria in different egg white models.

### Alkaline pH and CAMPs are key factors in the egg white bactericidal effect.

Bacteria in egg white are challenged with numerous antimicrobial peptides. However, the expression of two critical TCS systems involved in CAMP resistance—*phoPQ* and *pmrAB*—were downregulated significantly in egg white (Fig. 8A). This inhibition should result from the alkaline pH (9.3) of egg white, as both systems were induced and function at acidic pH (43, 44) and were inhibited at neutral or alkaline pH (43, 70 – 72). Thus, we hypothesized that the alkaline pH increases bacterial sensitivity to CAMPs by inhibiting the critical TCS systems *phoP/phoQ* and *pmrA/pmrB*, thereby reducing their ability to survive in egg white. Therefore, alkaline pH and CAMPs are key factors in the bactericidal effects of egg white.

How bacteria resisted CAMPs in an alkaline pH environment like egg white was unknown. It was postulated that pEtN and GlcUA modification of LPS, as well as decreasing the phosphate group of lipid A, were employed to decrease the negative charge of the bacterial surface, thereby reducing CAMP adsorption. In addition, protease oligopeptidase B was employed to degenerate the cytoplasmic CAMP.

### TCS *cpxR/A* and *sigE* play important roles in egg white resistance.

CpxR/A and SigE are critical regulators for the sensing and regulation of envelope stress in Gram-negative bacteria (45, 73), and both systems were significantly upregulated in egg white. During incubation, misfolded periplasmic proteins, damaged peptidoglycan structures, and alkaline pH can all activate the TCS CpxR/A (68, 74, 75). Activated CpxR exported extensive regulations to adapt to envelope stress and alkaline pH (Fig. 8B, see Data Sets S5 and S6 for details and references). The CpxR positively regulated itself (76, 77) as well as target genes involved in periplasmic space homeostasis (such as *htpX* and *spy*), peptidoglycan cross-linking and remodeling (*slt*, *ygaU*, *dacC*, and *amiC*) and envelope cross-linking (*ycfS*) to combat envelope stress caused by egg white. CpxR also has a direct and positive effect on the cation/antiporter *chaA* (78) and a negative effect on respiratory complexes (the *nuo* and *cyo* operons) (79). The result is to increase proton import and decrease export for egg white alkaline pH adaptation. In addition, CpxR induces expression of the ribosome modulation factor *rmf* and ribosome-associated inhibitor A (*raiA*) to inhibit translation (76).

CpxR also repressed SPI-1 genes by destabilizing its positive regulator, *hilD* (80). In addition, the major facilitator superfamily member *tsgA*, the esterase *yjfP*, and a putative membrane-bound redox modulator *alx* were also positively regulated by CpxR in egg white (78). Another seven unknown membrane or periplasmic space protein-encoding genes *yncJ*, *yjfN*, *yqjB*, *yebE*, *ytfK*, *yciX*, and *yceI* that belong to the Cpx regulon were also upregulated in egg white. How they contribute to egg white resistance is unknown. These results indicated that CpxR/A played a central regulatory role in the resistance of bacteria against egg white stress. Through gene deletion, we found that *cpxR* deletion abolished the survival ability of bacteria in egg white and proved a direct role for Cpx (Fig. 6).

The upregulation of *cpxR/A* as well as part of its regulon is consistent with previous studies (8). In our system, we found additional genes of the CpxR regulon that were significantly changed. These genes, including *slt*, *ygaU*, *ycfS*, *raiA*, *tsgA*, *alx*, *yqjB*, *yebE*, *ytfK*, and *yciX*, are involved in envelope cross-linking, translation, and unknown functions, indicating that there are different regulatory modes that bacteria use to survive in egg white compared with the bactericidal model.

We also found that *sigE* and *rseAB* were upregulated in egg white under our experimental conditions, which is different from the previous report (8). We found that 28 genes of the SigE regulon were upregulated significantly in egg white (see Data Set S6 for references). These genes include transcriptional regulatory genes *sigE*, *rseAB*, *rpoH*, *cpxR/A*, the LPS-related genes *eptB*, *lpxP*, the envelope cross-linking gene *tolA*, the DNA repair gene *recJ*, alkaline pH adaptation genes *yqjA* and *chaA*, the nitrogen metabolism genes *narI* and *hcp*, the amino acid metabolism genes *pdxA* and *asnB* as well as unknown *ytfJ* and *yqjB*. Upregulation of these genes indicates a role in cell envelope stress resistance, alkaline pH adaptation, and regulation of basal metabolism in egg white.

### Nutritional deprivation adaptation.

Nutritional deprivation through chelating factors is an important limiting factor for bacterial growth in egg white (9). Our results were highly consistent with previous reports (8) on increased iron uptake and biotin synthesis (Data Set S7) of *Salmonella* in egg white. This further supports the findings that iron and biotin limitation are important egg white antibacterial factors (11, 12, 21).

10.1128/mSphere.00700-18.7 DATA SET S7 Iron and biotin deficiency response. Download Data Set S7, XLSX file, 0.02 MB. Copyright © 2019 Huang et al. 2019 Huang et al. This content is distributed under the terms of the Creative Commons Attribution 4.0 International license.

### *Salmonella* pathogenicity island.

SPI-1 is involved in the invasion of nonphagocytic cells (81). The downregulation of SPI-1 genes indicates decreased invasion ability by exposure to egg white. SPI-2 plays an important role in survival in host cells by assisting nutrient acquisition, protecting the integrity of the vacuolar membrane, and facilitating its partitioning, also reducing the hydrolytic capacity of lysosomes (82). However, whether the upregulation of SPI-2 contributes to the survival of *Salmonella* in egg white is still unknown.

### Conclusions.

In summary, our results showed that *S.* Enteritidis employed multiple stress response mechanisms, such as SOS-dependent and SOS-independent DNA damage repair, alkaline pH adaptation, envelope repair especially for peptidoglycan biosynthesis, osmotic stress response, SPI-2, iron uptake, and biotin synthesis to combat the damages and limitations caused by the complex antimicrobial egg white. The *cpxR* and *sigE* regulator genes played central roles in coordinating bacterial metabolism to adapt to envelope damage and alkaline pH. After analyzing our RNA-Seq data, we proposed that bacterial osmotic stress in egg white was caused by the alkaline pH adaptation process and the energy-deficient state of bacteria in egg white and that the synergistic effect between alkaline pH and CAMPs is a key antibacterial factor of egg white. Also, two out of three DEGs that presented reduced survival rate at 37°C also worked at 20°C. Taken together, our results revealed resistance mechanisms that help bacteria to survive and persist in antibacterial egg white during egg storage.

## MATERIALS AND METHODS

### Bacterial strains and growth media.

A complete list of the bacterial strains used in this study is given in Table S1 in the supplemental material. A chicken meat isolate *S.* Enteritidis SJTUF10978 exhibiting strong viability in egg white was used for RNA-Seq analysis (83). M9 minimal medium supplemented with 2 mg/liter FeCl_3_·6H_2_O and microelements [10 μg/liter CuCl_2_·2H_2_O, 10 μg/liter Na_2_B_4_O_7_·4H_2_O, 10 μg/liter (NH_4_)_6_Mo_7_O_24_·10H_2_O, 40 μg/liter ZnCl_2_·4H_2_O, 10 μg/liter MnCl_2_·4H_2_O] (84) (M9FeS) was used. M9FeS or Luria-Bertani broth (LB) was routinely used in this study, and 37°C was the routine culture temperature without special instructions. Chloramphenicol (25 μg/ml) or ampicillin (100 μg/ml) was added when needed.

### Egg albumen preparation.

Fresh unfertilized specific-pathogen-free (SPF) eggs laid by 20- to 22-week-old chickens were purchased from Beijing Merial Vital Laboratory Animal Technology (Beijing, China) and were used within 2 days after laying. For every independent biological repeat, 50 SPF eggs were stored in a 37°C or 20°C or 4°C incubator with 65% relative humidity (RH) for 4 to 5 days as required. Then eggs were surface sterilized with 75% ethanol, and the egg white was separated under aseptic conditions from the yolk. The egg whites were pooled in sterile bags and homogenized with an Easymix (AES Chemunex, France) instrument for 8 min. The mixture was centrifuged twice at 12,857 × *g* for 5 min to remove the colloidal precipitate. The supernatant was transferred to a sterile bag and stored at 4°C for up to 5 days.

### Egg white antibacterial experiments.

Fresh bacteria (1 ml) of logarithmic phase was harvested, washed twice, and suspended in PBS buffer (10 mM Na_2_HPO_4_, 1.8 mM KH_2_PO_4_, 137 mM NaCl, 2.7 mM KCl [pH 7.4]) at room temperature. The bacterial suspension was adjusted by dilutions in PBS to reach 2.5 × 10^8^ CFU/ml and 2.5 × 10^4^ CFU/ml. Then 40-μl portions of bacterial suspensions were added to 160-μl portions of egg white in a Falcon 96-well microplate and mixed. This resulted in a final inoculation concentration from 5 × 10^7^ CFU/ml to 5 × 10^3^ CFU/ml and a final egg white concentration of 80% (vol/vol). The mixtures were then routinely incubated at 37°C (RH 65%) for 24 h. For Fig. 6B and C, the mixtures were incubated with egg white at 20°C and 4°C, respectively, for 5 days. The inoculation concentration and the final bacterial concentration after incubation were determined by dilution plate counts. The survival rate of each strain was calculated by the formula (final bacterial concentration after incubation/initial inoculum concentration). The relative survivability of each gene deletion mutant in egg white compared with the wild type was calculated by the formula (survival rate of each strain/survival rate of the wild type) and confirmed using two independent single clones with at least two biological repetitions at different times. For each biological repeat, three technical repeats were performed. Significance analysis was calculated using one-way ANOVA, and a *P* value of 0.05 was considered significant.

### Growth assays of bacteria cultured in egg white.

Single colonies of *S.* Enteritidis strains SJTUF 10978 and SJTUF 11463 were inoculated into liquid M9FeS medium and grown overnight at 37°C. The material was used to inoculate a 250-ml cone bottle containing 50 ml of M9FeS medium at a 1:50 ratio. The cells were cultured at 37°C with shaking at 200 rpm until mid-log phase. Samples (10 ml) were centrifuged at 5,000 × *g* for 3 min, washed twice, and suspended in PBS. The cell concentration was adjusted to 5 × 10^8^ CFU/ml, and 0.5 ml of cell suspension solution was added to 9.5 ml of PBS, mixed, and transferred to 40 ml of egg white in a 250-ml cone bottle that had been preheated to 37°C. This achieved a final inoculum concentration of 5 × 10^6^ CFU/ml and a final egg white concentration of 80% (vol/vol). These solutions were mixed and incubated at 37°C under a 65% humidified atmosphere. The inoculation concentration was determined by dilution plate counting. At 0.5, 1, 2, 4, 6, 8, 10, 12, 24, and 72 h after inoculation, 1-ml samples were taken to calculate the bacterial CFU/milliliter. Data from three independent biological replicates were used (mean ± SD). Student’s *t* test was used to analyze significant differences in cell viability of the resistant and sensitive strains at each time point.

### TEM observation.

Samples were centrifuged at 6,300 × *g* for 10 min at 4°C to collect bacterial precipitates and washed twice with 0.1 M phosphate buffer (PB) (68.4 mM Na_2_HPO_4_ , 31.6 mM NaH_2_PO_4_ [pH 7.2]). The samples were first fixed with 2.5% glutaraldehyde (>6 h) and then washed four times with 0.1 M PB for 15 min each time. The cells were postfixed with 1% OsO_4_ for 2 h and were again washed four times with 0.1 M PB for 15 min. The samples were dehydrated by a graded ethanol series (50%, 70%, and 90%) and dehydrated using 90% ethanol–90% acetone (1:1) and then transferred to absolute acetone. The dehydrated specimens were soaked in a 1:1 mixture of acetone and epoxy for 1 h and then in a 1:2 mixture of acetone and epoxy overnight. Finally, specimens were embedded in 100% epoxy, incubated for 7 h at room temperature, and then placed in an oven (65°C) for 48 h to polymerize. Finally, the prepared specimens were sectioned in an UC6 ultramicrotome (Leica, Germany) and stained with TI Blue for 10 min and then lead citrate for 6 min. The specimens were examined using a Tecnai G2 spirit Biotwin instrument (FEI, Hillsboro, OR).

### RNA isolation.

Bacterial culture and egg white inoculation were performed as described above in “Growth assays of bacteria cultured in egg white.” Bacteria were grown to mid-log phase in M9FeS, and 10 ml of culture was added to 4 ml (2/5 of the culture volume) of ice-cold RNA-stabilizing solution (5% [vol/vol] water-saturated phenol [pH 4.3] in 95% [vol/vol] ethanol) (85) immediately after harvest. The mixture was incubated on ice for 40 min, transferred to 2-ml RNase-free ice-cold centrifuge tubes, and centrifuged at 3,000 × *g* for 10 min at 4°C to pellet the bacteria. The cell pellets were then immediately frozen in liquid nitrogen and stored at −70°C. This sample was defined as the 0-h sample for the RNA-Seq experiments.

Four culture bottles of egg white incubation samples (50 ml/bottle) were harvested for RNA extraction by centrifuging at 12,857 × *g* for 2 min at 4°C in 50-ml Falcon tubes to pellet the bacteria, the supernatant was discarded, and tubes were inverted on paper for 15 s to remove the residual liquid as completely as possible. The tubes were placed on ice, and 1 ml of diluted ice-cold RNA-stabilizing solution (1.5% [vol/vol] water-saturated phenol [pH 4.3], 19% [vol/vol] ethanol in water) was immediately added to each tube to scatter the pellet. The pellets were transferred to 2-ml RNase-free centrifuge tubes, kept on ice for 40 min to inhibit the degradation of mRNA, and then centrifuged. The centrifugation step was repeated, and the samples were frozen in liquid nitrogen and stored at −70°C. At each time point, RNA from four parallel samples was pooled and taken as an independent biological replicate. Three independent biologically repeated experiments were performed at different times with different batches of SPF eggs.

RNA was extracted using TRIzol reagent (Invitrogen) according to the manufacturer’s instructions with the following modifications. Briefly, the stored samples were thawed on ice, resuspended in 100 µl TE buffer containing 50 mg/ml lysozyme (catalog no. 62970; Sigma), and incubated at 30°C for 10 min. TRIzol (1 ml) was added to the tubes, and the tube contents were repeatedly pipetted several times and vortexed vigorously for 1 min. The tubes were then centrifuged at 12,000 × *g* for 5 min at 4°C, and the supernatant was transferred to a new tube and incubated for 5 min at room temperature. Chloroform (0.2 ml) was then added, followed by 2- to 3-min incubation at room temperature. The sample was centrifuged for 15 min at 12,000 × *g* at 4°C, and the aqueous phase was extracted again with an equal volume of chloroform. The aqueous phases were combined, 0.5 ml isopropanol was added to each tube, and the tubes were placed at −70°C for 15 min. The tubes were then centrifuged for 30 min at 12,000 × *g* at 4°C, and the pellets were washed once with 75% ethanol. The RNA pellets were air dried at room temperature and then resuspended in 30 μl of RNase-free water. The purified RNA was stored at −70°C. RNA quality and integrity were verified by electrophoretic analysis and UV spectroscopy using NanoDrop and Agilent 2100 instruments. The RNA integrity numbers for all samples were between 8.2 and 9.7.

### Strand-specific transcriptome library construction and RNA sequencing.

The cDNA library preparation and Illumina sequencing were performed by BGI (Shenzhen, China). Briefly, 1 µg total RNA was digested with DNase I (Thermo Scientific) and treated with Ribo-Zero Magnetic Gold kit (Epicenter) to deplete rRNA. The protocol of TruSeq RNA Sample Prep kit v2 (Illumina) was used to construct the libraries. The RNA was fragmented into small pieces, and first-strand cDNA was generated using Super Script II (Invitrogen). The products were purified and used in a Second Strand Master Mix containing dATP, dGTP, dCTP, and dUTP. The purified fragmented cDNA was end repaired, purified, and A-tailed, and adaptors were attached. The purified product was digested with uracil-N-glycosylase to remove the dUTP-containing second-strand cDNA, followed by several rounds of PCR amplification to enrich the cDNA fragments. The PCR products were purified, and the libraries were assessed using the Agilent 2100 Bioanalyzer and qPCR. The qualified libraries were amplified on cBot to generate the flowcell cluster (TruSeq PE Cluster kit V3–cBot–HS; Illumina). The amplified flowcell was sequenced using the paired-end method on a HiSeq 2000 system (TruSeq SBS KIT-HS V3; Illumina). For every sample, more than 1 GB data were generated.

### Mapping of RNA-Seq libraries and differential gene expression analysis.

Sequencing reads containing sequencing adaptors and reads of low quality were removed to obtain clean reads. Clean reads were mapped against the reference genes of *S.* Enteritidis SJTUF10978 genome (GenBank accession number CP015524.1) and its plasmid pSJTUF10978 (accession number CP015525.1) with Bowtie2 (86), and the gene expression level was calculated by RSEM (87). The FPKM values incorporate normalization steps to ensure that expression levels for different genes and transcripts can be compared across runs. The DESeq2 method was used to calculate the differentially expressed genes (DEGs). Genes with a fold change ≥ 2, Bonferroni-corrected *P* value (*P*_adj_) ≤ 0.05 and FPKM ≥ 30 in at least one sample were determined to be DGEs. KEGG enrichment analysis of DEGs was carried out with the public database (http://www.geneontology.org/) and Kyoto Encyclopedia of Genes and Genomes (KEGG). Gene numbers were calculated for every term or pathway. In this study, significant pathways were identified as those with *P* values of <0.05, and the false-discovery rate (FDR) was set at <0.05. The Q value indicates the FDR of the specific pathway ,with lower values indicating greater significance.

### Quantitative RT-PCR assays.

To validate the RNA-Seq data, 18 to 21 genes were chosen for quantitative RT-PCR analysis. Removal of residual genomic DNA and cDNA synthesis was conducted using PrimeScript RT reagent kit with gDNA Eraser (catalog no. RR047A; TaKaRa) according to the manufacturer’s instructions. RNA integrity and the lack of residual DNA were confirmed by electrophoresis and the lack of a product of PCR amplification of the *gyrA* gene. The primers and their amplification efficiencies are listed in Table S2. Samples were run in triplicate and amplified using the SYBR Premix *Ex Taq* II reagent (TaKaRa, Dalian, China). The relative transcriptional level was determined using the 2^－^*^ΔΔCT^* threshold cycle (*C_T_*) method. The expression of 16S rRNA was used as an internal reference.

10.1128/mSphere.00700-18.10 TABLE S2 Primers for qRT-PCR, gene deletion, and complementation. Download Table S2, DOCX file, 0.02 MB. Copyright © 2019 Huang et al. 2019 Huang et al. This content is distributed under the terms of the Creative Commons Attribution 4.0 International license.

### Bacterial genetic manipulations.

Strains, plasmids, and oligonucleotide primers used are listed in Tables S1 and S2. The bacteriophage λ Red recombination system (26) was employed to construct gene deletion mutants of *S.* Enteritidis SJTUF10978, and suicide plasmid pRE112 (88) was employed to construct complementary strains. For the construction of gene deletion mutants, chloramphenicol-resistant knockout cassettes were generated by PCR amplification of pKD3 with primers that annealed with pKD3 at the 3′ end, and flanked by 45- to 50-nucleotide extensions that complemented the regions immediately adjacent to the region targeted for deletion at the 5′ end. After gel extraction, the PCR products were transformed into SJTUF 10978 competent cells that carried pKD46 and had been induced by 10 to 20 mM L-(+)-arabinose (Aladin) for 90 min before harvest. The DNA was introduced via electroporation (Bio-Rad MicroPulser, Hercules, CA) according to the manufacturer’s instructions. Shocked cells were rescued with 0.9 ml SOC medium and incubated for 2 h at 37°C before being spread onto chloramphenicol plates. After primary selection, the mutants were cultured in media lacking antibiotics at 42°C to remove pKD46. Isolates were colony purified once nonselectively at 37°C, and the mutations were verified by PCR (Table S2). Cm^r^ mutants were transformed with pCP20, and ampicillin-resistant transformants were selected at 30°C. A few colonies were purified once nonselectively at 42°C and then tested for loss of all antibiotic resistance. For the construction of complementary strains, the complementary suicide plasmids were constructed with pRE112 and PCR products of the gene and its upstream and downstream homology arms using suitable restriction enzyme sites in E. coli SM10 *λ pir*. After verification of sequencing, the correct plasmids were transformed into the corresponding SJTUF 10978 gene deletion mutants. Selection of single and double recombinations was performed by the method of Edwards et al. (88). The final deletion mutants and complementary strains were verified by PCR and sequencing (Table S2).

### Growth curve determination.

Growth curve measurement in LB and M9 medium of strain SJTUF 10978 (wild type) and its six gene deletion mutants and six complementary strains was performed at 37°C using Bioscreen C (OY Growth Curves, Finland). Three independent experiments were conducted.

### Accession number(s).

The final RNA-Seq data have been deposited in the Gene Expression Omnibus (GEO) database under accession no. GSE113880.
