# Supplementary figures and images for: Transcriptional Sequencing Uncovers Survival Mechanisms of Salmonella enterica Serovar Enteritidis in Antibacterial Egg White
Source: mSphere. 2019 Feb 13;4(1):e00700-18. doi: 10.1128/mSphere.00700-18 (PMC6374596; doi:10.1128/mSphere.00700-18)

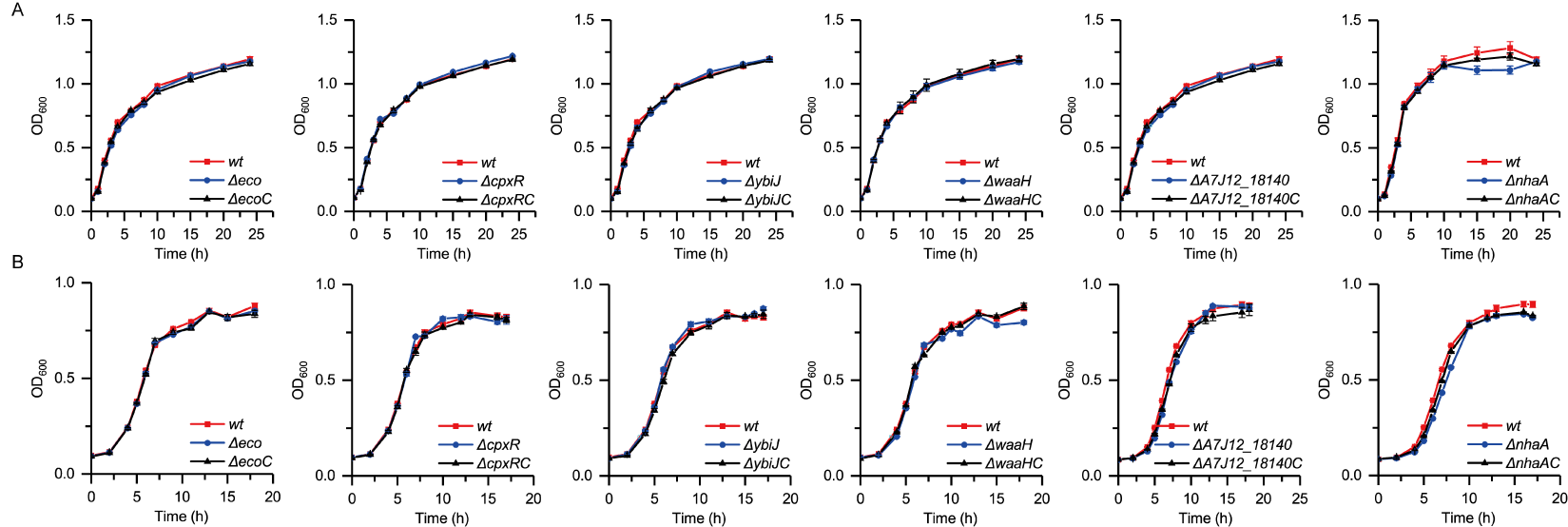

Supplement: FIG S1 [file mSphere.00700-18-sf001.tif]
