# Supplementary material for: Transcriptional Sequencing Uncovers Survival Mechanisms of Salmonella enterica Serovar Enteritidis in Antibacterial Egg White
Source: mSphere. 2019 Feb 13;4(1):e00700-18. doi: 10.1128/mSphere.00700-18 (PMC6374596; doi:10.1128/mSphere.00700-18)
Supplement: TABLE S1 [file mSphere.00700-18-st001.docx]

**Table S1 Strains and plasmids used in this study**

| **Strain and plasmid** | **Description or relevant genotype** | **Reference** |
| --- | --- | --- |
| SJTUF 10978 | *Salmonella* enterica Serovar Enteritidis strain, high survival ability in albumen | Laboratory stock |
| SJTUF 11463 | *Salmonella* enterica Serovar Enteritidis strain, low survival ability in albumen | Laboratory stock |
| SJTUF 10832 | *Salmonella* enterica Serovar Enteritidis strain, relative high survival ability in albumen | Laboratory stock |
| SJTUF 11501 | *Salmonella* enterica Serovar Enteritidis strain, relative high survival ability in albumen | Laboratory stock |
| ATCC 13076 | Salmonella enterica Serovar Enteritidis standard strain, | Laboratory stock |
| *Salmonella* Typhimurium ATCC 14028 | *Salmonella* Typhimurium standard strain | Laboratory stock |
| *Salmonella* Typhimurium SJTUF 10249 | Salmonella Typhimurium isolate | Laboratory stock |
| *Salmonella* Typhimurium SJTUF 10233 | Salmonella Typhimurium isolate | Laboratory stock |
| *Salmonella* Indiana SJTUF 12678 | *Salmonella* Indiana isolate | Laboratory stock |
| *E. coli* ATCC 25922 | *E. coli* | Laboratory stock |
| SJTUF 10978 *Δeco* | Deletion mutant of *eco* (*A7J12_12035*), encoding ecotin | This work |
| SJTUF 10978 *ΔecoC* | Complementary strain of *Δeco* | This work |
| SJTUF 10978 *ΔnhaA* | Deletion mutant of *nhaA* (*A7J12_23790*), Na^+^/H^+^ antiporter | This work |
| SJTUF 10978 *ΔnhaAC* | Complementary strain of *ΔnhaA* | This work |
| SJTUF 10978 *ΔybiJ* | Deletion mutant of *ybiJ* (*A7J12_03865*), response to DNA damage stimulus, function unknown | This work |
| SJTUF 10978 *ΔybiJC* | Complementary strain of *ΔybiJ* | This work |
| SJTUF 10978 *ΔA7J12_18140* | Deletion mutant of *A7J12_18140*, similarity to 60 kDa SS-A/Ro ribonucleoprotein homolog, function unkown | This work |
| SJTUF 10978 *ΔA7J12_18140C* | Complementary strain of *ΔA7J12_18140* | This work |
| SJTUF 10978 *ΔcpxR* | Deletion mutant of *cpxR* (*A7J12_20900*), response regulator of the two-component regulatory system CpxA/CpxR | This work |
| **Strain and plasmid** | **Description or relevant genotype** | **Reference** |
| SJTUF 10978 *ΔcpxRC* | Complementary strain of *ΔcpxR* | This work |
| SJTUF 10978 *ΔwaaH* | Deletion mutant of *waaH* (*A7J12_19115*), glycosyl transferase | This work |
| SJTUF 10978 *ΔwaaHC* | Complementary strain of *ΔwaaH* | This work |
| *E. coli* SM10 λ pir | thi thr leu tonA lacY supE recA::RP4-2-Tc::Mu Km λ pir, amplify plasmid pKD3 | Laboratory stock |
| plasmid pKD46 | Expresse bacteriophage λ Red recombinase | Presented by Professor Yao |
| plasmid pKD3 | Source for chloramphenicol acetyltransferase cassette | Presented by Professor Yao |
| plasmid pCP20 | Expresses FLP recombinase | Presented by Professor Yao |
| plasmid pRE112 | pGP704 suicide plasmid, *pir* dependent, *oriT*, *oriV*, *sacB*, Cm^r^ | (1) |
| pRE112*ecoC* | pRE112 derivative containing gene *eco* and its homologous arm fragment, cm^r^  constructed with SmaⅠand XbaⅠ | This work |
| pRE112*nhaAC* | pRE112 derivative containing gene *nhaA* and its homologous arm fragment, cm^r^  constructed with SacⅠand XbaⅠ | This work |
| pRE112*ybiJC* | pRE112 derivative containing gene *ybiJ* and its homologous arm fragment, cm^r^  constructed with SmaⅠand XbaⅠ | This work |
| pRE112*A7J12_18140C* | pRE112 derivative containing gene *A7J12_18140* and its homologous arm fragment, cm^r^  constructed with SmaⅠand XbaⅠ | This work |
| pRE112*cpxRC* | pRE112 derivative containing gene *cpxR* and its homologous arm fragment, cm^r^  constructed with SmaⅠand XbaⅠ | This work |
| pRE112*waaHC* | pRE112 derivative containing gene *waaH* and its homologous arm fragment, cm^r^  constructed with SmaⅠand XbaⅠ | This work |

**Reference**

1. Edwards RA, Keller LH, Schifferli DM. 1998. Improved allelic exchange vectors and their use to analyze 987P fimbria gene expression. Gene 207:149–157.
