# Supplementary material for: Transcriptional Sequencing Uncovers Survival Mechanisms of Salmonella enterica Serovar Enteritidis in Antibacterial Egg White
Source: mSphere. 2019 Feb 13;4(1):e00700-18. doi: 10.1128/mSphere.00700-18 (PMC6374596; doi:10.1128/mSphere.00700-18)
Supplement: TABLE S2 [file mSphere.00700-18-st002.docx]

| **Table S2 Primers for qRT-PCR, gene deletion** **and complementation** | | | |
| --- | --- | --- | --- |
| **Primer name** | **Sequence （5'-3'）*^a^*** | **Target gene** | **Application** |
| 18140F46 m | GTTGCAATAACTCTGGCATGGAGAATAACGGAAATGGGAAAAACAAGTGTAGGCTGGAGCTGCTTCG | *A7J12_18140* | Construction of gene *A7J12_18140* mutant via λ Red recombination |
| 18140R1600 | GGGAAAGATGTAATCCCGGCCAGCATTCGCGCCGCGCTGAATACTCTCATATGAATATCCTCCTTAG |  |  |
| 18140R981 | CTCTAGCTTCTAGACTAACTGCTCCTGCATTTCTTG |  | Construction of plasmid pRE112*a7j12_18140C* |
| 18140F893m | CGGGCCAGTTTTAGTTCATAG |  |  |
| 18140F206m | GCCACCGTTTTTCGCATC | *A7J12_18140* | Validation of the target gene mutation |
| 18140R1801 | CGCAACAAATGTAGATCCACAG |  |  |
| cpxRF49m | GATTAGCGACGCCTGATGACGTAATTTCTGCCTCGGAGGTACGTAAACAGTGTAGGCTGGAGCTGCTTC | *cpxR* | Construction of gene *cpxR* mutant via λ Red recombination |
| cpxRR712 | GTTAAACTTCCTATCATGAAGCGGAAACCATCAGATAGCCGCGACCACATATGAATATCCTCCTTAG |  |  |
| cpxRF988m | GCGAATAACGCACGACCAAC | *cpxR* | Construction of plasmid pRE112*cpxRC* |
| cpxRR1584 | CTCTAGCTTCTAGATTACGCGACATCACCAGCAG |  |  |
| cpxRF179m | GCCATAACAGCAGCGGTAACTT | *cpxR* | Validation of the target gene mutation |
| cpxRR838 | ATCAATCCCTGGCGCTGTT |  |  |
| ecoF45m | CGTGCATACCGAAAACAGCAGAAAGGAAATACCGTGAACAATCAAGTGTAGGCTGGAGCTGCTTCGA | *eco* | Construction of gene *eco* mutant via λ Red recombination |
| ecoR540 | CCGGCGTGTTCAGATGCCCGGTAAGTGTGACCGGGCAGCGTAACGCATATGAATATCCTCCTTAG |  |  |
| ecoF983m | GGCAACGTGTTCAACTGTGTG | *eco* | Construction of plasmid pRE112*ecoC* |
| ecoR912 | CTCTAGCTTCTAGACCCGTATAAGCCTGATTTTCCA |  |  |
| ecoF401m | CCATAACGACACGCTTCCATAG | *eco* | Validation of the target gene mutation |
| ecoR590 | GAGCGGATTTCCAGACGTGTT |  |  |

| **Primer name** | **Sequence （5'-3'）*^a^*** | **Target gene** | **Application** |
| --- | --- | --- | --- |
| nhaAF45m | TAAGATAGTAGATCCGAAGAATTCACCTGATTGAGAGAATAAAAAGTGTAGGCTGGAGCTGCTTCGA | *nhaA* | Construction of gene *nhaA* mutant via λ Red recombination |
| nhaAR1214 | AATGTCGCAAGGGAGCGATACATCGCTCCCCCTTTCCTTCGTACTGACATATGAATATCCTCCTTAG |  |  |
| nhaAF939m | GACTGGCTGCATATAAACCAGAG | *nhaA* | Construction of plasmid pRE112*mhaAC* |
| nhaAR2056 | CTCTAGCTTCTAGACTCGGCAAAAATCGCATGA |  |  |
| nhaAF172m | GTACGCGTAGCGGTCTATCATC | *nhaA* | Validation of the target gene mutation |
| nhaAR1361 | CCAGGGCTTTAATTTGACCAG |  |  |
| waaHF45m | CCACAGTATAAAGGCAGGGTAAATTAAGGTTTTTCTGGTAATCGTTGTGTAGGCTGGAGCTGCTTCG | *waaH* | Construction of gene *waaH* mutant via λ Red recombination |
| waaHR1082 | CAGTGGCAAGGCTGGGGGAAAATCGCCGCGCCTAAAAACGTGAGCGCTACATATGAATATCCTCCTT |  |  |
| waaHF851m | GCGCTGTCTTTCGATCTTGTC | *waaH* | Construction of plasmid pRE112*waaHC* |
| waaHR1991 | CTCTAGCTTCTAGAATAAGTTTTGCCTCAGTTTCGTC |  |  |
| waaHF133m | TCGCTAACGTAACGCTCTACTC | *waaH* | Validation of the target gene mutation |
| waaHR1147 | ACGCGCTTTTTCAGCGTC |  |  |
| ybiJF32m | CCATAAACAGAATAACCTGCGAGAGATTAATCATGAAAACCATTAAGTGTAGGCTGGAGCTGCTTCG | *ybiJ* | Construction of gene *ybiJ* mutant via λ Red recombination |
| ybiJR308 | TTCAAACAACAGCGATGTTACAGCCTGAAATCTGGCGGGCAGGTAAACATATGAATATCCTCCTTAG |  |  |
| ybiJF972m | GCGGAATGTCCCAGTGTGTA | *ybiJ* | Construction of plasmid pRE112*ybiJC* |
| ybiJR1192 | CTCTAGCTTCTAGAAGAATCATACCATCAAACTGACACC |  |  |

| **Primer name** | **Sequence （5'-3'）*^a^*** | **Target gene** | **Application** | |
| --- | --- | --- | --- | --- |
| ybiJF110m | TGAGCAAGGCCGTCAATTCTA | *ybiJ* | Validation of the target gene mutation | |
| ybiJR393 | GGGCAAATAAGGGTAACAAACA |  |  |  |
| PpKD3F951 | AAGTGATCTTCCGTCACAGGTAG | Localized in the cm^r^ fregment of pKD3 | Validation of the target gene mutation | |
| PpKD3R258 | CCATGGGCAAATATTATACGCA |  |  |  |
| **Primer name** | **Sequence (5'-3')** | **Target gene** | **Product size (bp)** | **Amplification efficacy** |
| 16SrDNAF580 | CGAATTAAACCACATGCTCCAC | *16S rDNA* | 167 | 1.01 |
| 16SrDNAR746 | CTGGTAGTCCACGCCGTAAAC |  |  |  |
| fljBF138 | AGGCCAGGCGATTGCTAA | *fljB* | 151 | 1.04 |
| fljBR288 | AGACAACTCACGCACACGCT |  |  |  |
| yafDF518 | ACCATAGCGGCCCTGTCA | *yafD* | 152 | 0.98 |
| yafDR669 | ATCCAGCGGTCGTCCAAA |  |  |  |
| recAF412 | GCGGTGGACGTCATTGTG | *recA* | 125 | 0.95 |
| recAR536 | AGCTTACGCATCGCCTGG |  |  |  |
| envZF334 | ATGGCGCAGCAATTAGGC | *envZ* | 154 | 1.04 |
| envZR486 | AACGGAAAAGCGGAGAAAAA |  |  |  |
| sefAF278 | CTGTCCCGTTCGTTGATGG | *sefA* | 203 | 1.01 |
| sefAR452 | GCTGGCAGGGTCGATTTAC |  |  |  |
| eptBF1136 | AGAACTCGCTTGCCCAGAAC | *eptB* | 157 | 0.99 |
| eptBR1292 | ATCATCTGCGCTTTCGTGC |  |  |  |
| clsAF838 | TGGGAAATCGAGACGGGTA | *clsA* | 116 | 0.98 |
| clsAR953 | GGAAAACCAGGACCGGAAG |  |  |  |
| lpxPF441 | GCCGATGATGGCGACCTAT | *lpxP* | 161 | 0.95 |
| lpxPR601 | GGTCGGGAGCAAACCAAA |  |  |  |

| **Primer name** | **Sequence (5'-3')** | **Target gene** | **Product size (bp)** | **Amplification efficacy** |
| --- | --- | --- | --- | --- |
| cirAF589 | GGCGTGCTGGGAATGAAA | *cirA* | 147 | 0.97 |
| cirAR735 | TGGTGTCCAGGCAAACTCG |  |  |  |
| iroBF386 | CCGTCGGCTTCGGTCATA | *iroB* | 241 | 0.96 |
| iroBR626 | AGACGCTTGCGATCAGGTG |  |  |  |
| iroNF750 | GGGCAATACCGGCTTTCA | *iroN* | 170 | 0.95 |
| iroNR919 | GGAAATCAGTGCGCTGTACG |  |  |  |
| proWF33 | ACAGGTGGCCGATACTACGA | *proW* | 150 | 0.95 |
| proWR182 | AGCGTCTTATGGAACGGGTC |  |  |  |
| proPF962 | GTTTCGGTCGACGTCCATT | *proP* | 127 | 0.99 |
| proPR1088 | ACCGCCAGCATCAACAAAC |  |  |  |
| waaHF302 | AGATGTATGAAACGCTGATGACC | *waaH* | 170 | 1.01 |
| waaHR471 | CAACGCCATACGCAACCA |  |  |  |
| cpxRF497 | ATTTGCTGGCGCAACACC | *cpxR* | 187 | 1.01 |
| cpxRR683 | ATCAGATAGCCGCGACCAC |  |  |  |
| ecoF258 | GGAAGGCTGGGGCTATGACT | *eco* | 152 | 0.95 |
| ecoR409 | TCGGCAGCTTGCTGTTGT |  |  |  |
| ycfSF426 | GGTGACGACCATTTCGGATAA | *ycfS* | 230 | 1.01 |
| ycfSR655 | GGATACAGCCGGAACTGACG |  |  |  |
| fimAF247 | CTGAATGACTGCGATCCGAA | *fimA* | 250 | 1.01 |
| fimAR496 | CGGTTGCCTTATAGCGTGC |  |  |  |
| pagCF161 | AAAGAATCGCCATCCTGAGTG | *pagC* | 177 | 0.95 |
| pagCR337 | CGGGTCTGTTGAGCCTGAA |  |  |  |

| **Primer name** | **Sequence (5'-3')** | **Target gene** | **Product size (bp)** | **Amplification efficacy** |
| --- | --- | --- | --- | --- |
| metEF916 | GCGCAGATTAACGCCATTGT | *metE* | 156 | 1.01 |
| metER1071 | CGCCAGTTCACCGCATTT |  |  |  |
| VirKF362 | TCTTGCTCGCTCACCTGGA | *virK* | 128 | 1.03 |
| VirKR489 | TAAACGTGCCAACGGCGTA |  |  |  |

*^a^*Letter with underline are homologous arm sequence immediately adjacent to the targeted region to be deleted
